# N1MΨU-modified mRNA vaccines break the mold in fish by enhancing innate immune activation

**DOI:** 10.1016/j.omtn.2026.102910

**Published:** 2026-03-20

**Authors:** Dean Porter, Luc Jouneau, Mathilde Peruzzi, Bertrand Collet, Bernard Verrier, Pierre Boudinot

## Main text

(Molecular Therapy: Nucleic Acids *37*, 102862; March 2026)

In the originally published article, the contributions of Quantoom and Pierre Libeau were noted in the acknowledgements in a way that may have been misinterpreted. Additionally, the methods used to design modified and unmodified mRNA vaccines were incorrectly noted in the materials and methods section, which may have cause some confusion. The text has now been clarified. The authors apologize for this inconvenience.

